# Amelioration of Huntington's disease phenotypes by Beta-Lapachone is associated with increases in Sirt1 expression, CREB phosphorylation and PGC-1α deacetylation

**DOI:** 10.1371/journal.pone.0195968

**Published:** 2018-05-09

**Authors:** Mijung Lee, Jae-Jun Ban, Jin-Young Chung, Wooseok Im, Manho Kim

**Affiliations:** 1 Department of Neurology, Seoul National University Hospital, Seoul, South Korea; 2 Department of Veterinary Internal Medicine and Geriatrics, College of Veterinary Medicine, Kangwon National University, Gangwon, South Korea; 3 Neuroscience Research Institute, Seoul National University College of Medicine, Seoul, South Korea; 4 Protein Metabolism Medical Research Center, College of Medicine, Seoul National University, Seoul, South Korea; National University of Singapore, SINGAPORE

## Abstract

Huntington’s disease (HD) is one of the most devastating genetic neurodegenerative disorders with no effective medical therapy. β-Lapachone (βL) is a natural compound obtained from the bark of the Lapacho tree and has been reported to have beneficial effects on various diseases. Sirt1 is a deacetylase of the sirtuin family and deacetylates proteins including the peroxisome proliferator-activated receptor gamma coactivator-1 alpha (PGC-1α) which is associated with mitochondrial respiration and biogenesis. To examine the effectiveness of βL on HD, βL was orally applied to R6/2 HD mice and behavioral phenotypes associated with HD, such as impairment of rota-rod performance and increase of clasping behavior, as well as changes of Sirt1 expression, CREB phosphorylation and PGC-1α deacetylation were examined. Western blot results showed that Sirt1 and p-CREB levels were significantly increased in the brains of βL-treated R6/2 mice. An increase in deacetylation of PGC-1α, which is thought to increase its activity, was observed by oral administration of βL. In an *in vitro* HD model, βL treatment resulted in an attenuation of MitoSOX red fluorescence intensity, indicating an amelioration of mitochondrial reactive oxygen species by βL. Furthermore, improvements in the rota-rod performance and clasping score were observed in R6/2 HD mice after oral administration of βL compared to that of vehicle control-treated mice. Taken together, our data show that βL is a potential therapeutic candidate for the treatment of HD-associated phenotypes, and increases in Sirt1 level, CREB phosphorylation and PGC-103B1 deacetylation can be the possible underlying mechanism of the effects of βL.

## Introduction

Huntington’s disease (HD) is a progressive brain disorder caused by the expansion of a CAG repeat in the huntingtin gene. This mutation results in the production of the polyglutamine expanded huntingtin protein (mHtt), leading to involuntary choreiform movements, cognitive impairment, and neuropsychiatric symptoms [1, 2]. The length of the CAG repeat of the huntingtin gene is closely related to the age of onset and severity of the disease [3, 4]. Although the exact mechanism of the disease progression has not been elucidated, mHtt causes transcriptional dysregulation, which can lead to neuronal cell death in the brain [5, 6]. In addition, recent reports suggested that mitochondrial dysfunction induced by mHtt contributes to the progression of HD [7–9]. Many defects in mitochondria have been observed in various HD mouse models, cell models and patients, and striatum is the brain region that is particularly vulnerable to mitochondrial impairment [10].

The quinone-containing compound, β-Lapachone (3,4-dihydro-2,2-dimethyl-2H-naphthol[1,2-b]pyran-5,6-dione; βL) is a natural product derived from the roots of the south American Lapacho tree (*Tabebuia avellanedae*) [11]. βL has been known to have a variety of medicinal properties such as anti-cancer, anti-inflammatory, anti-bacterial, anti-viral and wound healing effects [12–15]. Since βL exhibits anticancer effects in various cancer cell lines, a clinical trial is underway to test it as a candidate for anticancer drugs [16, 17]. βL has also an anti-inflammatory effect on neuronal cells, and thus it has a potential as a therapeutic agent for neurological diseases caused by excess inflammation [18, 19]. In addition, βL induces apoptosis through the increase of mitochondrial reactive oxygen species (ROS) in cancer cells [20–22], while it has an anti-oxidative effect in other cell types including neuronal cells [23]. Although βL has been reported to have various beneficial effects, its effectiveness on motor and mitochondrial impairments of HD model have not been investigated yet.

Sirt1 is the most important and best characterized sirtuin involved in gene expression, cell cycle regulation, DNA repair, stress response, apoptosis, neurogenesis, metabolism, and aging [24–28]. Sirt1 mediates chromatin condensation by deacetylation of histones [29]. In addition, Sirt1 deacetylates non-histone substrates including nuclear transcription factors, retinoic acid receptor, and peroxisome proliferator-activated receptor gamma coactivator-1 alpha (PGC-1α) [30–32]. mHTT aggregates in the HD cells directly interacts with and inhibits Sirt1 deacetylase activity, affecting multiple downstream targets [33, 34] and decrease transcription of genes that modulates mitochondrial functions [10], antioxidant defense [35], and neurotrophic support [36]. Recent evidence also indicates that PGC-1α is a potent inducer of mitochondrial biogenesis and is involved in HD pathogenesis and neurodegeneration [37]. PGC-1α is activated by deacetylation by Sirt-1 [38–41], and activation of PGC-1α rescues cells from oxidative damage through mitochondrial biogenesis and activation [42, 43]. It is also believed that phosphorylated CREB is the predominant regulator of PGC-1α [43, 44]. Mitochondrial dysfunction, which is caused by dysregulation of PGC-1α induced by mHtt, plays an important role in the progression of HD, and improvement of mitochondrial function and HD phenotypes by restoration of PGC-1α has been reported by previous studies [45, 46]. Thus, the activation of PGC-1α by Sirt-1 and CREB phosphorylation can be a potential target for the treatment of HD.

In the present study, we investigated whether βL affects behavior phenotypes associated with HD, including the rota-rod performance impairment and clasping behavior. Furthermore, its effects on brain-derived Sirt1, PGC-1α acetylation status, and phosphorylated CREB level were examined. Primary neuronal cells from R6/2 mice shows mitochondrial dysfunction along with mHtt aggregation and can be used as an *in vitro* HD model. This HD model was used to show the change of mitochondrial superoxide level by βL treatment.

## Materials and methods

### Ethics statement

All animal experiments were performed in accordance with the National Institutes of Health guide for the care and use of laboratory animals (NIH Publications No. 8023, revised 1978). All animal studies were approved by the Institutional Animal Care and Use Committee at Seoul National University Hospital.

### Animal model and oral administration of β-lapachone

We use transgenic mice of the R6/2 line (B6CBA-Tg(HDexon1)62Gpb/3J, 111 CAGs) and their WT littermates (Jackson Laboratories, USA). The R6/2 transgenic mouse model is the most widely used animal model of HD and expresses exon 1 of a human mHtt. These mice were obtained by crossing ovarian transplant hemizygote females with B6CBAF1/J males. The mice were housed in groups with ad libitum access to food and water and a 12 hours light/ 12 hours dark cycle. The genotype was assessed using a PCR assay, and CAG repeat length was measured by DNA sequencing (Macrogen, Seoul, South Korea) The average number of CAG repeat was 166 ± 1.1 (n = 4, S1 Fig).

Considering protective effect of βL, oral-injection was started at 5 weeks old. Dosage was set at 70 mg per body weight (kg) and calculated by the body weight measured just before the experiment. βL was dissolved in 0.1% sodium lauryl sulfate (SLS) solution at a concentration of 7 mg/ml for oral gavage, and oral administration was performed by sonde for once a day during 6 weeks. Same volume of 0.1% SLS was used for vehicle control group.

### Rota-rod and clasping test

The Rota-rod test was conducted using a rota-rod apparatus (Jungdo Instruments, Korea). Mice were placed on the rod with an accelerating rotating speed from 4 to 40 rpm over a period of 3 minutes with a 15 minutes rest between trials. Mice were trained on three consecutive days for three trials per day at 4 weeks of age. Three trials were performed and the mean latencies to fall were used to analyze data. Rota-rod evaluation was performed every week from 5 to 11 weeks. Clasping test is the behavior test to evaluate motor impairment of HD transgenic mice and the motor functions of the mice were tested using clasping test. In each session, the mouse was suspended by the tail for 30 sec and the time during which the hindlimbs are entirely retracted and touching the abdomen was measured. Clasping test was performed at 11 weeks of age.

### Protein extraction and western blot

Brains of R6/2 mice were isolated at 11 weeks of age, immediately frozen on liquid nitrogen, and stored at -80°C until protein extraction. Protein was extracted using RIPA buffer (Radioimmunoprecipitation assay buffer, Thermo Scientific, USA) containing freshly added protease inhibitor and phosphatase inhibitor (Roche Diagnostic Systems, USA). The protein content was determined using a BCA protein assay kit (Bicinchoninic acid assay, Pierce, USA). Thirty micrograms of protein samples were separated on sodium dodecyl sulfate-polyacrylamide gel electrophoresis (SDS-PAGE) and transferred onto polyvinylidene fluoride membrane (Millipore, USA). Membranes were blocked with 5% non-fat dried milk dissolved in 1× TBST (Tris-buffered saline with 0.1% v/v Tween-20) for 1 h at room temperature. Blots were then incubated at 4°C overnight with primary antibodies diluted as recommended in the manufacturer's instructions. The following primary antibodies were used: anti-Sirt1 (1:200; Santa Cruz, SC-15404, CA, USA), p-CREB (1:1000; Cell signaling, 9198S, MA, USA), CREB (1:1000; Cell signaling, 9104, MA, USA) and anti-β-actin (1:200; Santa Cruz, SC-47778, CA, USA). Blots were then incubated with horseradish peroxidase-conjugated secondary anti-mouse or anti-rabbit antibodies (1:3000, GE Healthcare, NJ, USA), and developed using ECL solution (Enhanced chemiluminescenece solution, Advansta Inc., CA, USA). Between each incubation step, the membrane was washed three times with 1× TBST. ImageJ software was used for obtaining intensity values of the blots.

### *In vitro* HD model

We developed an *in vitro* HD model by isolating neuronal stem cells (NSCs) from R6/2 HD mice [47, 48]. To culture primary NSCs, mice at postnatal day 3 were sacrificed by decapitation. For the subventricular zone dissection, brain was sliced into 2 mm coronal sections in PBS with 0.6% glucose, pH7.4 at 4°C using scissor and forceps. Brain slice was washed with PBS/glucose and digested in TrypLE express enzyme (Thermo Scientific, USA) for 10 minutes. Digested tissues were filtered through 40 μm nylon mesh filter (Falcon, UK), and then washed three times. Live cells were calculated using the trypan blue dye method and seeded at 1 x 10^5^ cells/ml in 25 cm^2^ flask. NSCs were incubated in culture medium consisting of DMEM/F12, 1% PSA (penicillin-streptomycin-amphotericin; Invitrogen, USA), 2% B27 Supplement (Gibco BRL, USA), 10 ng/mL EGF (Invitrogen, USA) and 10 ng/mL bFGF (Invitrogen, USA) at 37°C in a 95% O_2_, 5% CO_2_ humidified atmosphere. For MitoSOX red analysis, NSCs were differentiated in the differentiation medium, which was composed of DMEM/F12, 1% PSA, 2% B27, and 5% FBS. Cultured neurospheres reaching 150 μm diameter were transferred to sterile tissue culture tubes and spun at 1,000 rpm for 5 min. Neurosphere pellets were resuspended with differentiation medium. Approximately ten neurospheres were then transferred into individual 24-well tissue culture plates containing poly-L-lysine-coated cover glass.

### PGC-1α acetylation assays

PGC-1α lysine acetylation was analyzed by immunoprecipitation of PGC-1α followed by western blot using acetylated-lysine antibody (1:1000; Cell Signaling, 9681S, MA, USA). Tissue protein extracts and protein content were prepared using RIPA buffer and BCA protein assay kit. For immunoprecipitation, 500 μg of protein lysates samples were incubated at 4°C rotation overnight with anti-PGC1α (2 μg; Santa Cruz, SC-13067, CA, USA) diluted as recommended in the manufacturer's instructions. Purified proteins were separated by 8% SDS-PAGE and immunoblotting using anti-PGC1α (1:200; Santa Cruz, CA, USA) and acetylated-lysine (1:1000; Cell Signaling Technology, USA). The supernatant and precipitated protein was immunoblotted with PGC-1α and actin antibody to confirm the immunoprecipitation process (S2 Fig).

### MitoSOX red analysis

For *in vitro* HD model NSCs were differentiated in a 24-well plate for 3 days. For MitoSOX Red (Invitrogen, USA) staining, cells were washed once with PBS (WelGene, Korea) and stained with MitoSOX Red for 15 min at 37°C. Cells were washed again with PBS and analyzed by flow cytometry (FACS Cailbur, BD Biosciences, USA) and image with fluorescence microscopy (BX61, Olympus Corporation, Japan). The FACS and fluorescence was analyzed by with Winmdi 2.9, or captured by fluorescence microscopy according to the manufacturer’s instructions.

### Statistical analysis

All values indicated in the figures are presented as mean ± standard error mean (SEM), and results of western blot, flow cytometry and clasping test were analyzed using Student's *t*-test. Rota-rod test was analyzed using one-way ANOVA. The data were analyzed using Prism software (GraphPad, USA), and a 2-tailed probability value below 0.05 was considered statistically significant.

## Results

### β-Lapachone increases Sirt1 and CREB phosphorylation and PGC-1α deacetylation

**Fig 1:**
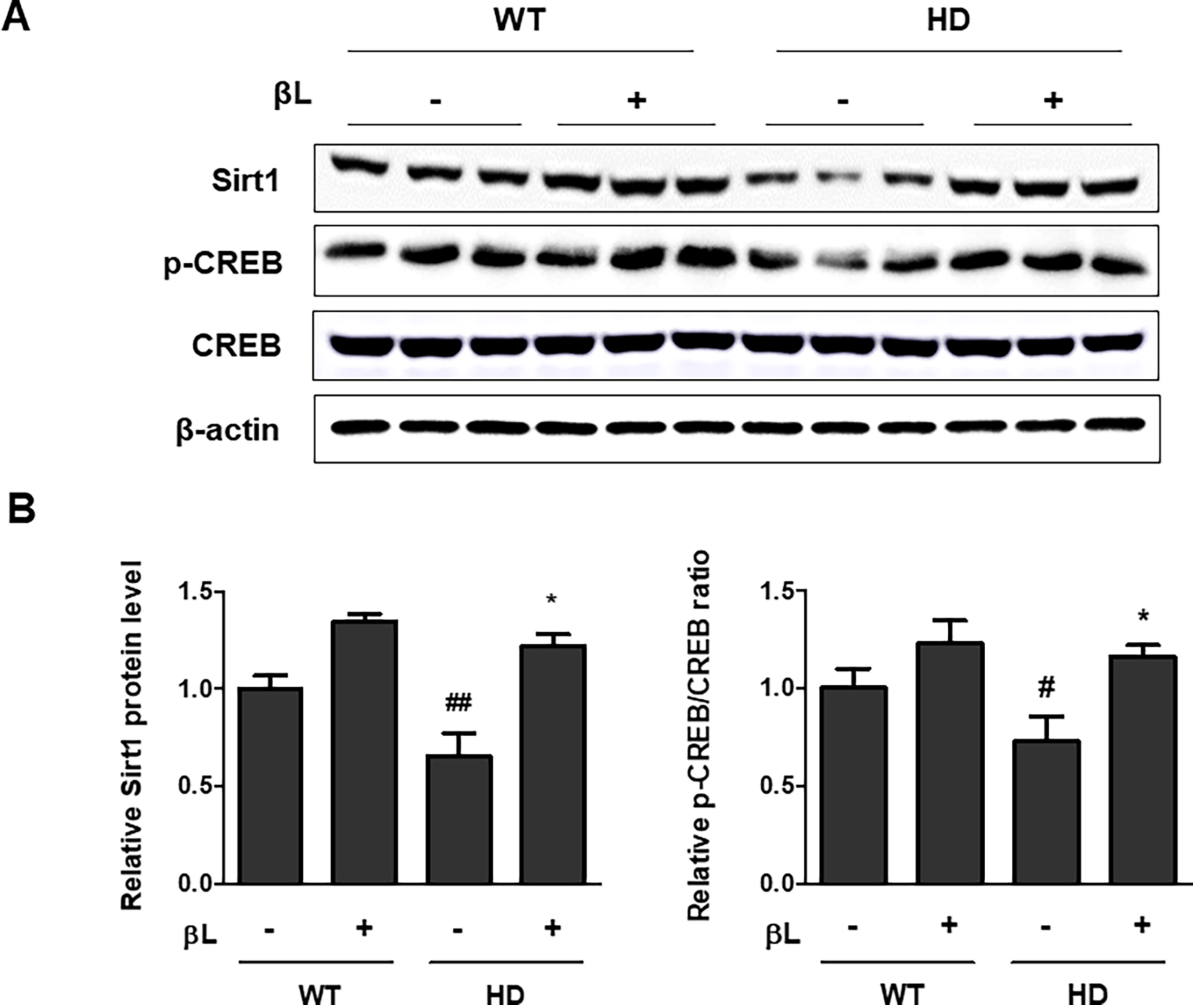
Increase in Sirt1 and p-CREB levels by oral administration of βL in mice. βL was orally administered to R6/2 mice and wild type littermates for 6 weeks from 5 to 11 weeks of age and total brain proteins were extracted. Total proteins were probed with antibodies to examine Sirt1, p-CREB, and total CREB levels of the brain by immunoblot assay. Western blot results showed three representative protein samples (A). Relative Sirt1 level and p-CREB/CREB ratio were normalized with β-actin and represented as a bar graph (B). *p < 0.05; significant differences compared to the vehicle-administered R6/2 mouse. #p < 0.05 and ##p < 0.01; indicate significant differences compared with the WT control.

**Fig 2:**
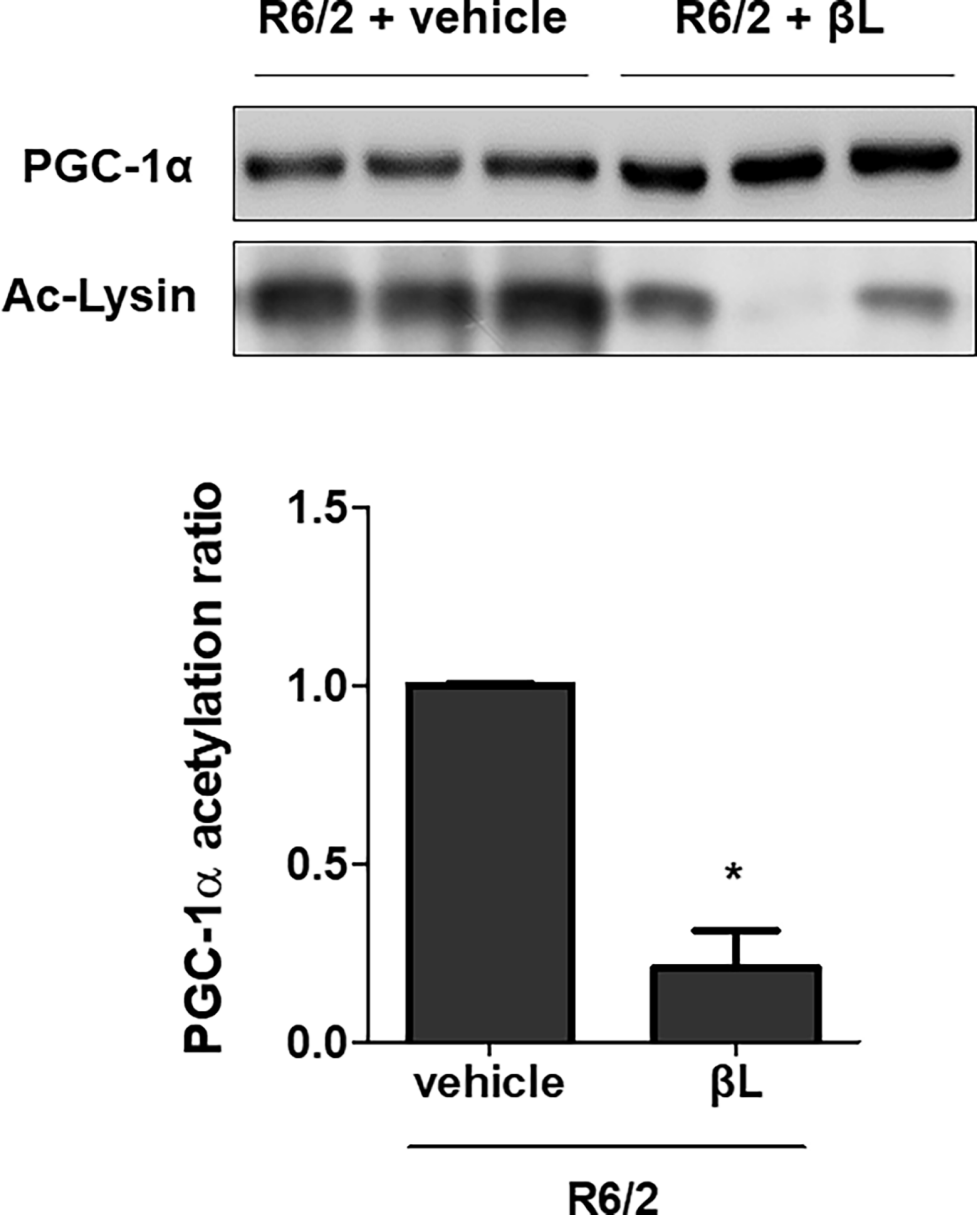
Reduction in PGC-1α acetylation by βL administration in mice. βL was orally administered to R6/2 mice for 6 weeks from 5 to 11 weeks of age. After the sacrifice, brains were isolated and an immunoprecipitation was performed using the PGC-1α antibody. Immunoprecipitated proteins were separated by SDS-PAGE and blotted with anti-PGC-1α and anti-acetylated-lysine antibodies to measure the level of PGC-1α acetylation. Relative band intensities were analyzed using the Image J software. The acetylation level of PGC-1α was analyzed by the intensity of acetylated lysine relative to the amount of total PGC-1α (lower panel). The graph shows means ± SEM. *p < 0.01 indicates significant differences when compared to the control group.

To examine the effects of βL on Sirt1 and CREB phosphorylation and PGC-1α acetylation, βL was orally administered to R6/2 mice for 6 weeks from 5 to 11 weeks of age. Brains of R6/2 mice administered with βL or the vehicle were isolated and protein levels were analyzed using western blot. Treatment with βL promoted the expression of Sirt1 and p-CREB in the brain of R6/2 mice (Fig 1). To confirm the change in the acetylation status of PGC-1α in the brain, total PGC-1α was immunoprecipitated and acetylated lysine level of PGC-1α was measured. The results show a reduction in PGC-1α acetylation in the brain following βL treatment (Fig 2). These data indicate that oral injection of βL can increase the level of Sirt1, p-CREB and deacetylation of PGC-1α in the brain of HD mice.

### β-Lapachone ameliorates mitochondrial superoxide level in an *in vitro* HD model

**Fig 3:**
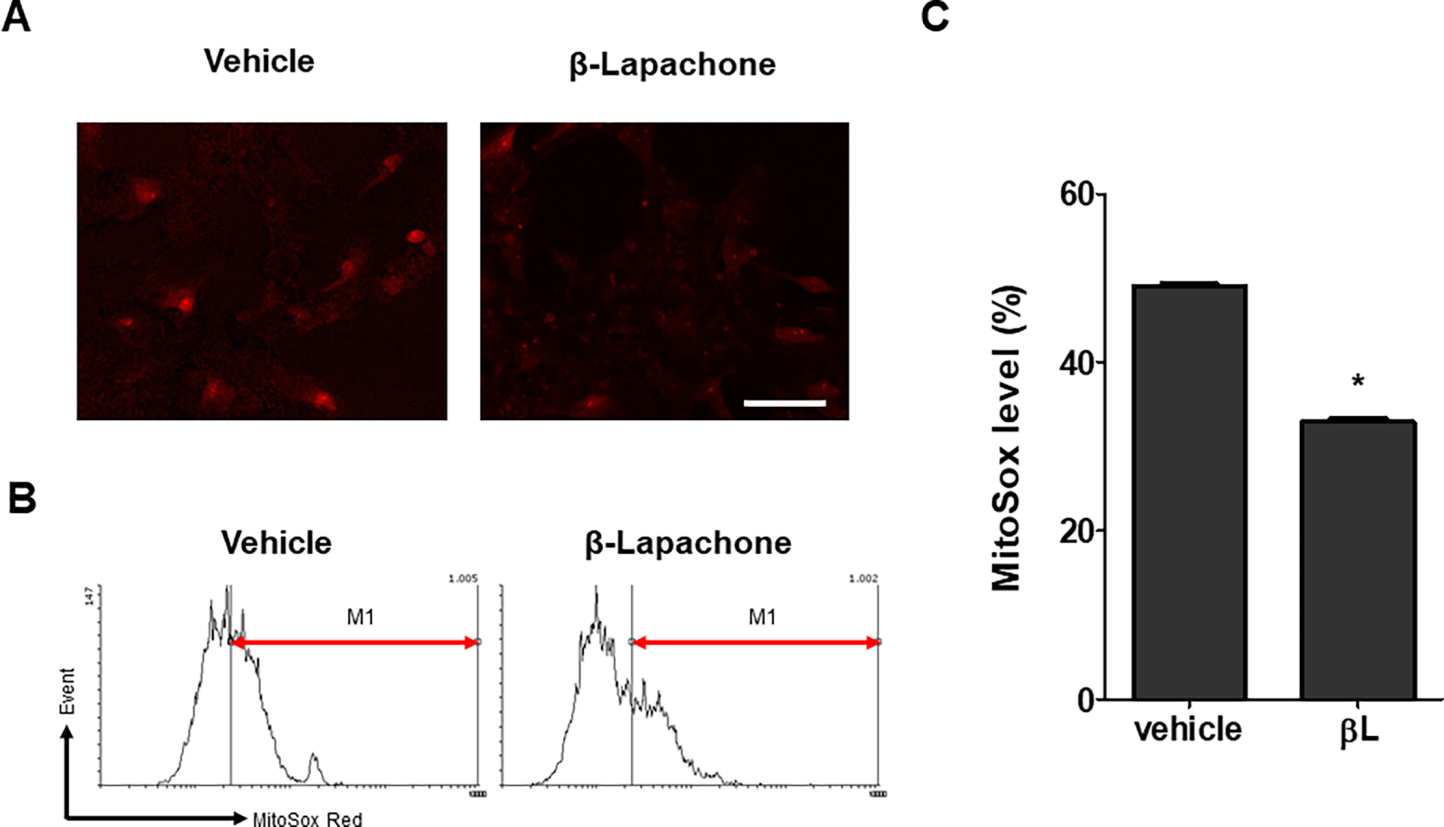
Decrease in mitochondrial superoxide level by βL treatment of an *in vitro* HD model. R6/2 mouse-derived NSCs were cultured and differentiated in a differentiation medium. After differentiation, βL was added for 3 days and mitochondrial superoxide levels were measured by immunocytochemistry (A) and flow cytometry (B) after MitoSOX red staining. The red, roundish objects are individual cells with MitoSOX staining, and the M1 marker of the flow cytometry result indicats a Cy3-positive population. The percentage values of NSCs with M1 after vehicle or βL treatment are represented in a bar graph (n = 3) (C). The graph shows means ± SEM. *p < 0.05 indicates significant differences when compared to the vehicle-treated group.

To investigate whether βL has a mitochondrial protective role in a HD cell model, we treated an *in vitro* HD model with βL and mitochondrial superoxide level was measured. For the *in vitro* HD model, NSCs were isolated from the brains of R6/2 HD mice and culture by the neurosphere method. HD NSCs could be differentiated in a growth factor-deprived medium and showed mitochondrial dysfunction and mHtt aggregation *in vitro* [47, 48]. Our MitoSOX red result showed that treatment with βL tended to reduce the red fluorescence intensity compared to that in the vehicle-treated group, indicating that mitochondrial superoxide levels were reduced by the βL treatment (Fig 3A). Flow cytometry result also confirmed the amelioration of mitochondrial superoxide levels by βL (Fig 3B and 3C). These data show that βL can prevent mitochondrial dysfunction in an *in vitro* HD model.

### β-Lapachone attenuates behavioral phenotypes of R6/2 HD mice

**Fig 4:**
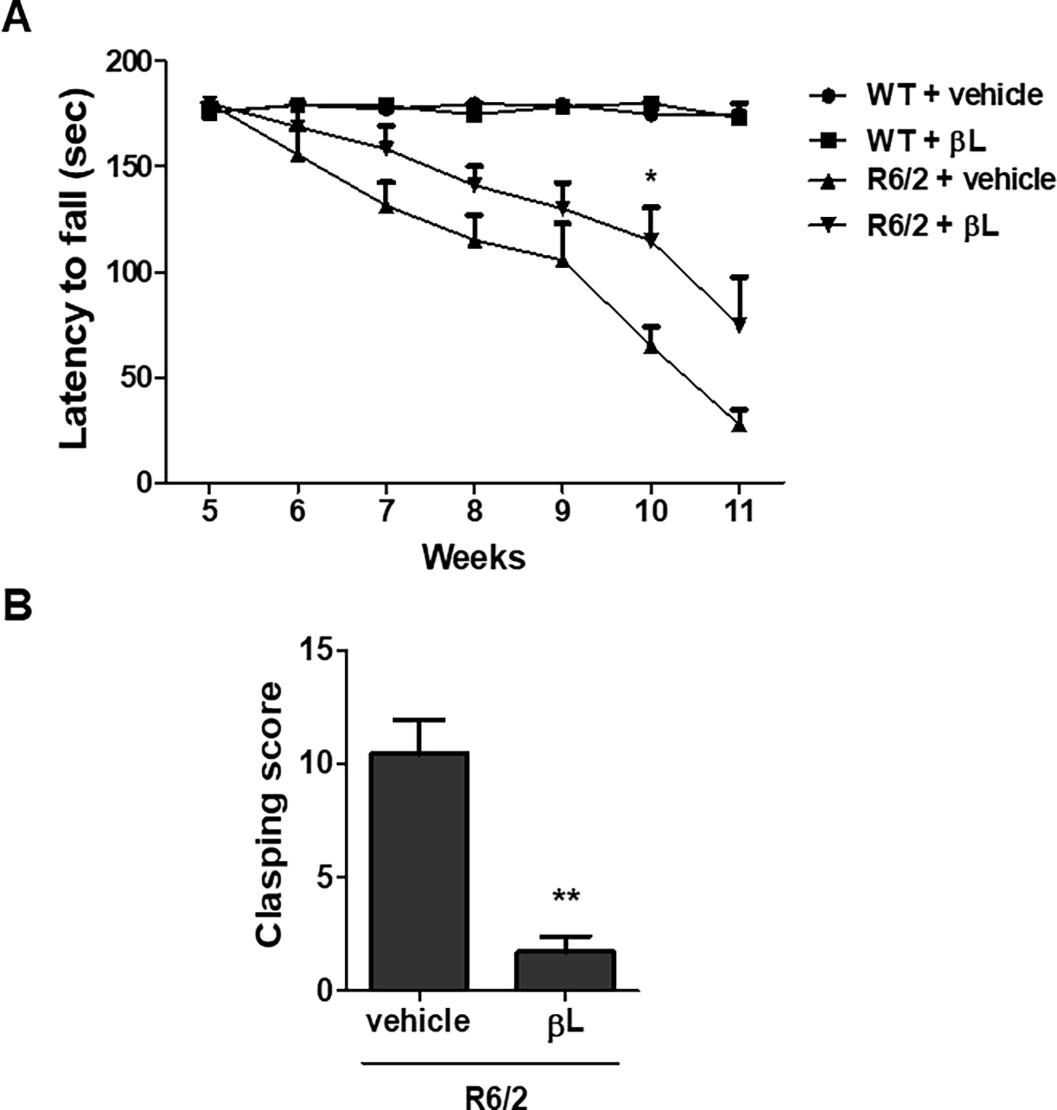
Behavioral improvement of R6/2 mice by oral administration of βL. To examine the changes in behavior phenotypes, βL was orally administered to female wild-type or R6/2 mice at 5 to 11 weeks of age and rota-rod performances were measured every week (n = 5 per group) (A). Clasping tests were performed at 11 weeks of age (n = 10 per group) (B). The graph shows means ± SEM. *p < 0.05 and **p < 0.001 indicates significant differences compared to the vehicle-treated R6/2 mice.

Our data show that R6/2 mice exhibit impairments in the rota-rod performance from 5 to 11 weeks of age, whereas the motor performance was maintained in WT littermates. Next, we administered βL to female R6/2 mice from 5 to 11 weeks of age and rota-rod performance was examined. R6/2 mice injected with βL showed overall longer mean latencies to fall with a statistically significant difference at 10 weeks of age compared to control R6/2 mice administered with vehicle (Fig 4A). We also measured the clasping score at 11 weeks of age to further validate the motor function impairment of HD. R6/2 mice administered with βL had a lower clasping time than the vehicle-administered group (Fig 4B). Collectively, our data show that oral administration of βL could attenuate the progression of HD-associated motor phenotypes along with increases in Sirt1, p-CREB, and PGC-1α deacetylation.

## Discussion

Collectively, our results demonstrate that βL could increase Sirt1, p-CREB, and PGC-1α deacetylation in the brains of R6/2 HD mice. In addition to these molecular changes, we observed decreased mitochondrial superoxide levels in *in vitro* HD neuronal cells upon βL treatment. We also found that behavioral deficits in performance on rota-rod and clasping test were improved in βL-administered R6/2 mice.

Sirt1 is an evolutionarily conserved protein with NAD^+^-dependent deacetylase activity and it plays a role in cellular metabolism [49]. It is also believed that Sirt1 has a neuroprotective role in various degenerative neurological disorders [50]. Activation of Sirt1 in HD also results in decreased neuronal cytotoxicity mediated by mHtt, and ablation of Sirt1 exacerbates mHtt-induced toxicity [33, 34]. This beneficial effect is believed to be due to the deacetylase activity of Sirt1. mHtt interacts with Sirt1 to inhibit the deacetylase activity and the regulation of its downstream target genes [51]. Given the importance of this deacetylase activity of Sirt1 in HD, our results suggest that βL may increase Sirt1 and modify HD and other neurodegenerative diseases.

The transcription factor CREB is known as a major regulator of PGC-1α transcription. CREB expression and phosphorylation is a potent survival signal for neuronal cells [52]. Mice with an impaired CREB response element resulted in progressive striatal neurodegeneration and clasping behavior, which are key features of HD [53]. Recent evidence also indicates the involvement of PGC-1α in progression and neurodegeneration of HD. PGC-1α is reported to fine-tune energy homeostasis, metabolism, and mitochondrial function in response to specific needs [37, 54]. Metabolic abnormalities and oxidative damage in HD appear to be associated with dysfunction of PGC-1α activity by mHtt [45, 55]. The HD brain expresses low-level PGC-1α target genes and dysregulation of PGC-1α exhibits phenotypes such as hyperactivity, clasping, and striatal neurodegeneration [56, 57]. The elevated activity of PGC-1α can increase the expressions of enzymes that protect brain cells from oxidative damage induced by ROS, and CREB activity is essential for these beneficial roles of PGC-1α [43]. In addition, Sirt1 interacts with and deacetylates PGC-1 α, leading to activation of PGC-1α [39]. Thus, activation of PGC-1α by deacetylation and CREB phosphorylation might account for moderate behavioral improvement and molecular benefits, such as amelioration of mitochondrial ROS, of the βL treatment. Since the current results is the observation of the change at the single time point, it cannot be assured that PGC-1α deacetylation and CREB phosphorylation occurred sequentially after the change in Sirt1. To investigate the role of Sirt1 in modulating CREB and PGC-1α activations, knock-down or siRNA experiments should be performed in future studies.

βL derived from the Lapacho tree is a potential novel anticancer agent currently under clinical trials [16, 17], although its mechanism of action is still unknown and needs to be elucidated. βL induces ROS-induced cell death in U87 glioblastoma, pancreatic cancer cells, and bacteria [20, 22, 58]. On the other hand, βL was reported to exhibit antioxidant and anti-inflammatory effects in neuronal cells via up-regulation of antioxidant enzyme expressions or NRF2/ARE signaling [19, 23]. In addition, βL was reported to have anti-melanogenic effects, suggesting that βL may be a potential depigmentation agent [59]. These contradictory results from previous studies suggest cell type specific responses to βL. In addition to the differences in the recipient cell type, treatment dose may also vary between one study to another. Although experiment to determine the extent of βL penetration into the brain has not been performed, βL can directly or indirectly modulate central nervous system [19, 60]. In our *in vitro* primary NSC model, βL showed a decrease in superoxide level but no difference in JC-1 assay, which is an experiment to confirm mitochondrial membrane potential (S3 Fig). However, considering the beneficial role of increased Sirt-1, PGC-1 and CREB phosphorylation on mitochondria, βL is likely to mitigate the mitochondrial dysfunction seen in *in vitro* neuronal model of HD.

Although there was an improvement of rota-rod performance in female mice, no difference of rota-rod score were shown in male mice (S4 Fig). Despite the exact reason for this gender difference is unknown, it can be assumed that this is presumably due to differences in gender-specific rota-rod ability. In case of amyotrophic lateral sclerosis mouse model, male transgenic mice shows motor dysfunction earlier than female mice with no gender differences in other specific behavior tests [61]. In our result, clasping test and western blot analysis showed no gender differences between male and female mice.

The aggregation of mHTT in the HD neuronal cells is the most representative phenotype of HD and is considered to be the cause of the disease. Although immunohistochemistry result of βL-treated mice showed no difference in mHtt aggregates and positive cell number compared to wild-type, the aggregation spots tended to be smaller than in some mice treated with βL (S5 Fig). Since previous studies reported that PGC-1α induction could reduce the mHtt aggregates in the cortex [46] and βL reduced the formation of polyglutamine aggregation by promoting autophagy and Sirt1 in SH-SY5Y cells [62], it is possible that βL may modify the mHtt aggregates formation in R6/2 HD brains. However, the precise effect of βL on mHtt aggregates formation need to be further investigated at multiple time points of disease progression through various methods.

In summary, βL attenuates mitochondrial superoxide level in an *in vitro* HD model and behavioral phenotypes of R6/2 HD mouse model along with promotion of Sirt1 expression, CREB phosphorylation and deacetylation of PGC-1α. Our *in vitro* and *in vivo* data provide evidence for the therapeutic potential of βL in alleviating HD-associated symptoms.

## Supporting information

S1 Fig Genomic DNA sequencing of R6/2 mouse. The tail of the R6/2 mice was cut and genomic DNA was isolated for DNA sequencing. The red arrow indicate the beginning and end of the CAG repeat.
(TIF)

S2 Fig Identification of PGC-1α in immunoprecipitated protein. Total brain lysates was immunoprecipitated with anti-PGC-1α antibody. The supernatant and precipitated protein was immunoblotted with PGC-1α and actin antibody.
(TIF)

S3 Fig JC-1 staining of HD in vitro model. HD neuronal cells were treated with or without βL at 24h after seeding. Cells were subjected to JC-1 staining at 48h after treatment, and data showed no significant change by βL treatment (n = 3 each).
(TIF)

S4 Fig Rota-rod result of R6/2 male mice. βL was orally administered to male R6/2 mice at 5 to 11 weeks of age and rota-rod performances were measured every week. No significant change was observed between vehicle and βL group.
(TIF)

S5 Fig Immunohistochemistry of mHtt aggregation in βL or vehicle-administered R6/2 mouse brain. The brains of R6/2 mice administered vehicle or βL were isolated and mHtt aggregation was examined using immunohistochemistry of EM48 staining. βL group showed tendency of mHtt aggregate size to be small in cortex and striatum. Size bar = 100 μm.
(TIF)
