# Supplementary figures and images for: Amelioration of Huntington's disease phenotypes by Beta-Lapachone is associated with increases in Sirt1 expression, CREB phosphorylation and PGC-1α deacetylation
Source: PLoS One. 2018 May 9;13(5):e0195968. doi: 10.1371/journal.pone.0195968 (PMC5942716; doi:10.1371/journal.pone.0195968)

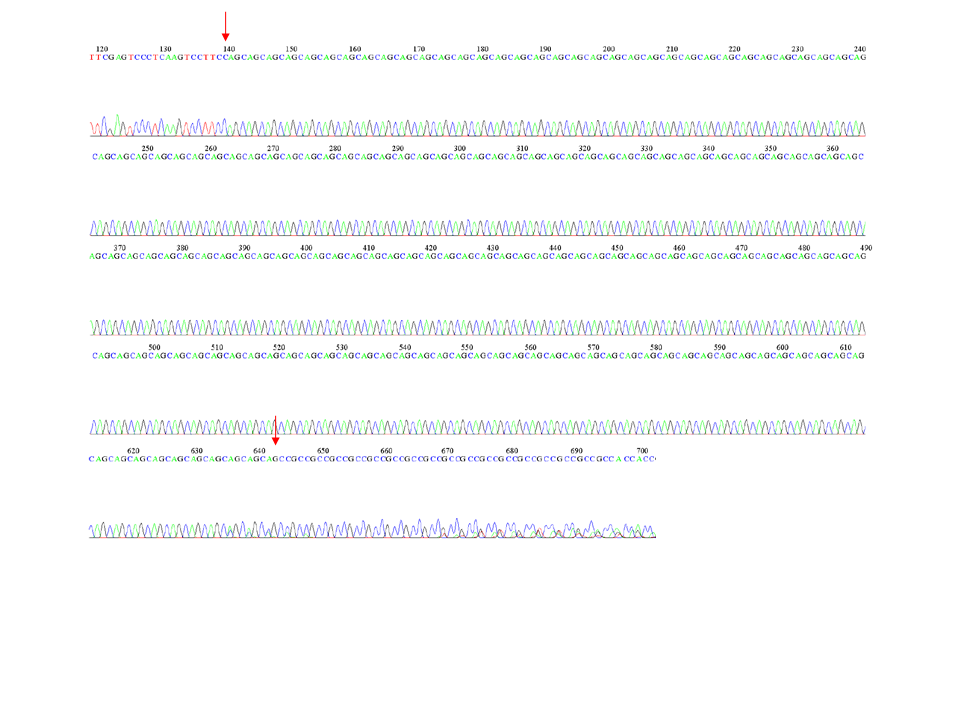

Supplement: S1 Fig — The tail of the R6/2 mice was cut and genomic DNA was isolated for DNA sequencing. The red arrow indicate the beginning and end of the CAG repeat. (TIF) [file pone.0195968.s001.TIF]

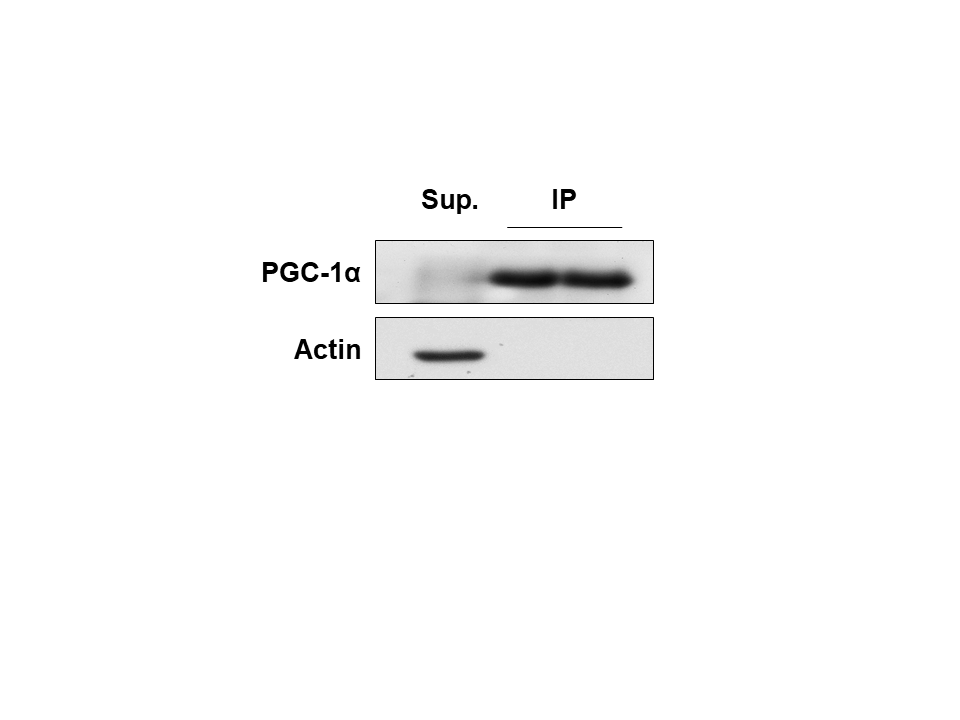

Supplement: S2 Fig — Total brain lysates was immunoprecipitated with anti-PGC-1α antibody. The supernatant and precipitated protein was immunoblotted with PGC-1α and actin antibody. (TIF) [file pone.0195968.s002.TIF]

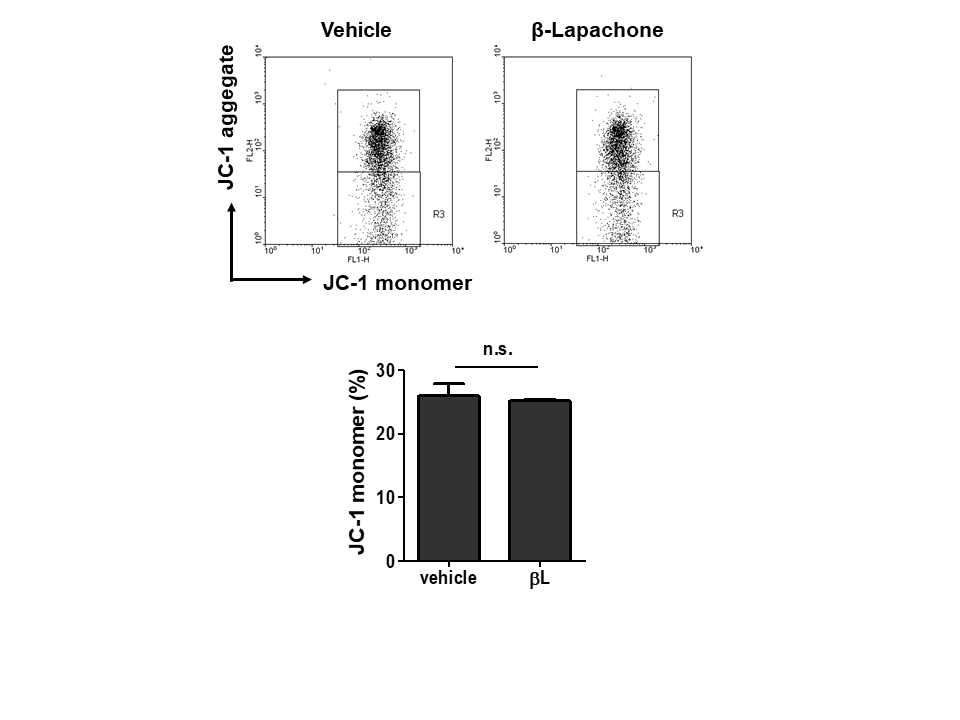

Supplement: S3 Fig — HD neuronal cells were treated with or without βL at 24h after seeding. Cells were subjected to JC-1 staining at 48h after treatment, and data showed no significant change by βL treatment (n = 3 each). (TIF) [file pone.0195968.s003.TIF]

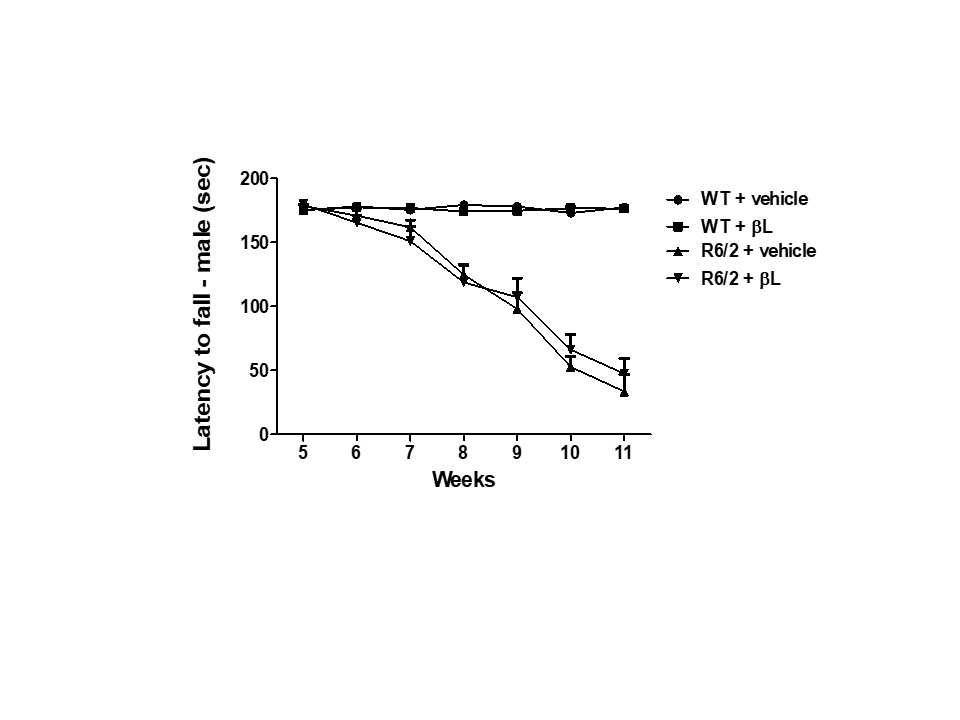

Supplement: S4 Fig — βL was orally administered to male R6/2 mice at 5 to 11 weeks of age and rota-rod performances were measured every week. No significant change was observed between vehicle and βL group. (TIF) [file pone.0195968.s004.TIF]

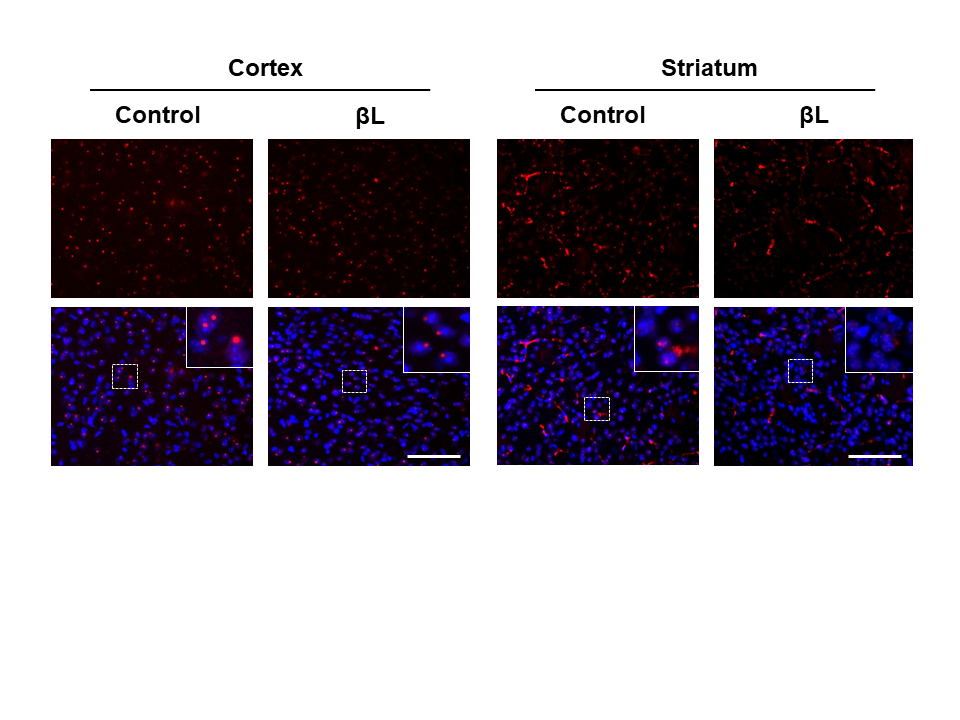

Supplement: S5 Fig — The brains of R6/2 mice administered vehicle or βL were isolated and mHtt aggregation was examined using immunohistochemistry of EM48 staining. βL group showed tendency of mHtt aggregate size to be small in cortex and striatum. Size bar = 100 μm. (TIF) [file pone.0195968.s005.TIF]
